# Integrated multimorbidity management in primary care: why, what, how, and how to?

**DOI:** 10.15256/joc.2016.6.95

**Published:** 2016-11-01

**Authors:** Joachim P. Sturmberg, Richard J. Botelho, Bruno Kissling

**Affiliations:** ^1^School of Medicine and Public Health, The University of Newcastle, Callaghan, NSW, Australia; ^2^Department of Family Medicine, University of Rochester, Rochester, NY, USA; ^3^Novant Health, Charlotte, NC, USA; ^4^Institute of Family Medicine, Medical Faculty, University of Bern, Bern, Switzerland

**Keywords:** multimorbidity, primary care, health system, health care reform, integrated health care systems, complex adaptive health care systems, physiological networks, philosophy of health, nonlinear dynamics, somato-psycho-socio-semiotic model of health

## The epidemic of multimorbidity

Policymakers regard “the epidemic of multimorbidity” as the greatest threat to the sustainability of healthcare systems. They believe the solution is “integrated care”, “*The search to connect the healthcare system (acute, primary medical and skilled) with other human service systems (e.g. long-term care, education and vocational and housing services) in order to improve outcomes (clinical, satisfaction, and efficiency)”* [1]. This definition includes key characteristics of complex adaptive systems. People act as agents who evolve in their characteristics and behaviours over time. These agents constantly learn and adapt in real time to changing contexts. These systems display emergent dynamic non-linear behaviours resulting from ongoing iterative feedback amongst their agents.

Emergent outcomes do not have linear “cause and effect” relationships and can best be understood in hindsight. Emergent behaviours are highly sensitive to context; consequently, the “same” approach used by different agents in different contexts will not produce the same outcomes. Agents navigate toward mutually agreed outcomes by constantly adapting to evolving changes within the context of local constraints [2].

A complex adaptive system approach overcomes many of the dysfunctions in the current health systems, in particular the fragmentation of patient care [3]. Overcoming fragmentation requires continuous adaptation to changing circumstances – a constant challenge for patients, health professionals, community service providers, and policymakers.

How can the already overburdened primary healthcare services achieve these goals?

To address the complex challenge, we first must reflect on three key questions:

- What is health?
- What is disease?
- What is multimorbidity?

## Reflections on health, disease, and multimorbidity

Definitions of health are contested [4–6]. Health is a personal experience rather than an objective state. The subjective experience of health arises from the complex adaptive interactions from four sources: our body, mind, social context, and our sense-making processes about our experiences. The question, “How do you rate your health in general on a scale of ‘excellent’, ‘very good’, ‘good’, ‘fair or poor’?”, captures a person’s health experience and closely correlates with future morbidity and mortality [7–9].

Diseases are socially constructed and re-constructed. For example, lowering “normal” blood sugar thresholds labels more people “diabetic”, and the creation of a new category between normal and abnormal blood sugar levels results in people being labelled “pre-diabetic” [10–12]. Notwithstanding labelling effects and ageing, most people remain unaffected by diseases throughout their life: about 10% of 65–74-year olds have five or more diseases, rising to 20% in 75–84-year olds, and to just over 30% in those aged over 85 years [13,14]. Similarly, the number of diseases does not correlate with health perceptions. The majority of aged people enjoy good health despite their multimorbidities (77% of 65–74-year olds, 70% of 75–84-year olds, and 63% of those aged >85 years) [15].

**Figure 1:**
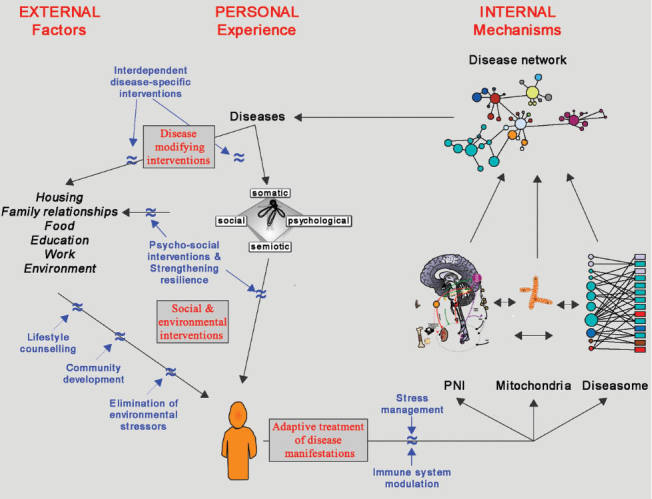
A network model of multimorbidity. The figure illustrates the network relationships between external and internal factors on the personal experience of health. Interventions that modify the person’s health and health experience are highlighted. PNI, pscyhoneuroimmunology.

Viewed from a complex adaptive system perspective, the experience and clinical aspects of multimorbidity result from interconnected physiological disturbances of genomic [16], metabolomic [17,18], autonomic [19], and immunological network interactions [20,21]. Furthermore, an individual’s internal coping mechanisms and external environment both modulate physiological function and affect the person’s experience of health and illness (Figure 1 and Box 1) [22].

**Box 1 Health, disease, and multimorbidity: summary points.**

**Health**

- Is a subjective state
- Is influenced by many external factors
- Good health perception is associated with lower mortality and lower health service use

**Disease and multimorbidity**

- Principally defined by doctors/bureaucrats
- An inevitable feature of the life trajectory
- Is non-linearly distributed across age brackets, i.e. most people are not affected
- Most people stay healthy for longer, and most have a short period of disability before dying
- Most people experience “good health” most of the time, independent of their morbidities

## Appreciating healthcare through a complex adaptive system lens

The way we think reflects the way we see the world [23]. The way we view health, disease, and multimorbidity shapes how we act. Health professionals are acculturated [24] in institutions that view health through the prism of disease. They are disease managers, not optimizers of people’s health, regardless of morbidities. Consequently, health professional–patient encounters are predominately disease-centric, and seldom focus on the person or the person’s experience of health.

Adopting a complex adaptive system approach to health, disease, and multimorbidity recognizes the importance of managing the patient’s quality of life as much as their diseases. This approach considers how medical interventions improve the patient’s quality of life or detract from it, despite being “current best practice”. This process explores the impacts of physical, social, and emotional functions on the patient’s changing experience of health.

## Designing complex adaptive health systems

Given our deeper understanding of how the context of a person’s life impacts his/her health, we have to redesign primary care to enable it to provide integrated care. Properly implemented integrated care addresses all of a person’s emerging needs to achieve and maintain a good health experience.

Systemic problems require systemic solutions. Health systems must design adaptive healthcare organizations that respond to their patients’ changing needs (see NEJM Catalyst; catalyst.nejm.org). Using bottom-up approaches, engage all stakeholders to deliberate on designing integrated services. This calls for systemic change in the approach to redesigning organizations. It requires addressing four questions asking why, what, how, and how to? The systemic redesign of organizations requires them to address the following issues [25,26]:

- Purpose (Why?)
- Specific goals (What?)
- Shared values (How?)
- “Simple rules” (How to?).

Purpose and goal questions define the overall and specific objectives of an organization, and its shared values shape its culture. “Core values” remain stable in a constantly changing world. Values clarify what the organization is and articulate what it stands for. Values create a culture of safety and trust for learning together. They guide behaviours and interactions and influence the quality of personal and professional relationships.

Understanding purpose, goals, and shared values is pivotal in order to define a set of three to five “simple rules” (or operating principles) that guide the actions and behaviours within an organization. Statements regarding purpose, goals, values, and “simple rules” have an important function – they act as a reference point for decision-making and resolving unavoidable conflict; determining which options are most aligned with purpose, goals, values, and “simple rules”.

## System malalignments

Specialists define their purpose and goals as managing organ-specific diseases (e.g. heart disease, kidney disease, or diseases of the nervous system). In contrast, general practice/family medicine views its role as optimizing “personal health experiences”. General practitioners (GPs)/family physicians (FPs) focus on minimizing patients’ illness experiences despite their multimorbidities. Minimizing patients’ illness experiences involves addressing their interconnected physical, social, emotional, and sense-making needs in resource-constraint environments.

Divergent purposes and goals are a characteristic in pluralistic societies. They interfere with designing complex adaptive health systems and organizations for integrated care based on health equity.

Many other entities, while providing important inputs to the delivery of care to individuals and communities, legitimately pursue different goals. The self-interests of corporations (pharmaceutical and device industry, for-profit health and indemnity insurers) and disease-­focused advocacy groups drive their specific agendas that can run counter to a person-centred approach to integrated care.

## Workshop feedback

The purpose, goals, values and “simple rules” framework were discussed with 80 GPs/FPs from across the world at the WONCA Europe conference workshop in Copenhagen, Denmark, June 2016. The group regarded the divergent purpose and goal statements amongst the different stakeholders as the root cause for the dysfunction of their health systems.

These doctors agreed with the person-focused purpose and goal statements for the health system. They shared remarkably similar views about the values that guide their approaches to patient care. Based on the experience of workshop participants, five “simple rules” to deliver integrated multimorbidity care were developed (Table 1):

**Table:** 

Rule 1.	Develop ongoing trustful relationships with patients, their families, and their care team
Rule 2.	Understand patients’ and their families’ experiences, needs, and preferences
Rule 3.	Enhance patients’ and their families’ capabilities to manage their own health and their diseases
Rule 4.	Explore with patients and their families the impact of treatments on their future health
Rule 5.	Engage with the community to enhance social networks and health-promoting infrastructures.

## The future of integrated multimorbidity management

GPs/FPs feel up to the challenges posed by their patients with multimorbidities. However, they are frustrated by the fragmented approach to multimorbidity management. Guidelines focus on individual disease and neglect to:

- Take into account the patient’s social and environmental context and the interactive effects between morbidities
- Enhance the patient’s and their family’s abilities to manage the demands of treatment regimens
- Address the psychosocial impacts of illness on the patient and their family.

To optimize integrated care, we must go beyond guidelines [27] and connect the healthcare system with other human service systems in order to improve all relevant outcomes [1]. We need to do the following:

- Develop a complex adaptive healthcare system that puts the patient and their family at the centre of care [1,26,28–31]
- Train physicians capable of treating patients’ multimorbidities as pathophysiological network dysfunctions
- Educate health professionals to recognize and manage the patient’s illness
- Activate health professionals to engage in building health-promoting communities.

The workshop participants want a public discourse on how to:

- Put the needs of the patient at the centre of the health system
- Present a realistic picture about the nature of health and illness, the roles of self-care and medical care, and the abilities, limitations, and harms of biomedical interventions
- Lead the practice-level changes required to create the time and space to manage patients’ needs in all its dimensions (embracing colocation of health and social services)
- Implement a network-thinking approach to manage patients’ illnesses and diseases.

## Leading health service and health system redesign

The current pessimism and discontent with fragmented multimorbidity care has emerged as a catalyst for a ­bottom-up movement to health service and health system redesign. Health professionals increasingly appreciate the interdependencies among the personal, emotional, social, and sense-making processes as the basis to optimize the health of patients with multimorbidity. We must advocate for the essential political, social, and environmental changes needed to optimize the health of patients with multimorbidity.

The “WONCA Special Interest Group Complexities in Health”^1^ supports all health professionals to develop the necessary complex adaptive system skills for delivering person-centred integrated health (and social) care. This entails broadening the agenda to make health, notwithstanding multimorbidities, the center of a redesigned health system.

## Figures and Tables

**Table 1:** The four core principles that achieve a complex adaptive organization: summary points.

Core principle	Summary of points for consideration or action
Define **purpose** – why do we work together (Why?)	“Managing patients’ illnesses” “Illness” is the experience of “loss of health”, in the presence or absence of identifiable disease
Define **goals** – what do we want to achieve (What?)	“Helping people to regain their good health experiences” In the presence or absence of identifiable disease
Define **values** – how we behave (How?)	Key values mentioned during the workshop: See the person, not the disease Have a dialogue with the patient Help the person to feel healthy/experience health The general practitioner/family physician is the guide to the person’s health Empower the person in looking after their own health Involve the community in improving people’s health experiences Avoid care fragmentation Explore how the patient copes with his illness and disease Integrate care with other health professionals and the community
Define “**simple rules**” – how do we act and interact (How to?)	Five “simple rules” for person-/family-/community-centred care Develop ongoing trustful relationships with their families and their care team Trust allows for the emergence of the most adapted management strategies that take into consideration the interactive effects of evidence-based guidelines, polypharmacy, and specialist opinions Understand the patient’s and their families’ experiences, needs, and preferences How do the patient’s interdependent personal, familial, social, and environmental circumstances impact their current health experience? How to negotiate an individualized approach for managing the patient’s multimorbidity within their family, social, and community context? Enhance patients’ and their families’ capabilities to manage their own health and their diseases Does the patient require additional medical, psychological, nursing, social, environmental, and/or economic support? Explore with patients and their families the impact of treatments on their future health How might medications, investigations, procedures, and referrals positively as well as negatively impact on the patient’s health, well-being and disease states? Encourage patients to take their time to consider/reconsider their goals, expectations, and ability to adapt to changing health experiences Engage with the community to enhance social networks and health-promoting infrastructures Which additional health services would improve patient care and well-being? Which community services/infrastructure could promote disease prevention and well-being of those with morbidities

## Footnotes

1 http://www.globalfamilydoctor.com/groups/SpecialInterestGroups/ComplexitiesinHealth.aspx.
